# OLDER ADULT'S SEPSIS CARE: CROSSING THE CONTINUUM: LESSONS LEARNED, OPPORTUNITIES PRESENTED

**DOI:** 10.1093/geroni/igac059.1806

**Published:** 2022-12-20

**Authors:** Teresa Cranston, Joseph Kontra, Lanyce Roldan

**Affiliations:** Penn Medicine Lancaster General Health, Lancaster, Pennsylvania, United States; Penn Medicine Lancaster General Health, Lancaster, Pennsylvania, United States; Penn Medicine Lancaster General Health, Lancaster, Pennsylvania, United States

## Abstract

Sepsis is the body’s overwhelming response to infection that can lead to tissue damage, organ failure, and death. Delays in identification and treatment are associated with increased morbidity and mortality especially for older adults. It is key that clinicians practice collaboration and communication when diagnosing & treating patients that may have a different presentation. The Continuous Monitoring Unit (CMU) consists of RNs, who monitor the Sepsis Dashboards 24 hours/7 days a week. These nurses evaluate all BPA data points. They dismiss the irrelevant and align the significant while looking for possible notes of infection. They act upon the BPA when the patient meets criteria. They collaborates with the provider to initiate recommended sepsis care. Once the sepsis diagnosis is confirmed, the CMU RN communicate with inpatient nursing via EMR shift reporting and Care planning tools. Nursing utilizes these tools for daily rounds with the health care team communicating patient discharge needs. Identifying the Sepsis survivor on admission allows for early sepsis survivor education, discharge preparation and outpatient follow up referrals. Case Management utilizes an electronic application for outpatient referrals and includes a Sepsis Survivorship alert. Community resources, alert to a sepsis diagnosis will follow up within 24-48 hours of discharge to insure that the patient is having a smooth transition. Referred providers can escalate care to the PCP for early intervention therefore reducing worsening state or readmission. Specifically, our home care agency monitors the patient closely upon discharge due to the sepsis survivor’s tenuous state and reinforces sepsis education.

